# Effects of Monochromatic Light on the Development of Immune Organs, Antioxidant Capacity and Immune Response of Thymus and Bursal of Fabricius in Yangzhou Geese

**DOI:** 10.3390/ani16010037

**Published:** 2025-12-23

**Authors:** Yiyi Cheng, Binbin Guo, Yingqing Xu, Jie Liu, Wen Yang, Yunxiang Zhang, Yujuan Zhang, Jiying Liu, Huanxi Zhu, Gang Luo

**Affiliations:** 1 Jiangsu Key Laboratory of Sericultural and Animal Biotechnology, School of Biotechnology, Jiangsu University of Science and Technology, Zhenjiang 212100, China; chengyiyi1027@163.com (Y.C.); xyingqing233@163.com (Y.X.); yangwen9901@163.com (W.Y.); yunxxzhang23@163.com (Y.Z.); zhangyujuan907@163.com (Y.Z.); liujiying@just.edu.cn (J.L.); 2 Key Laboratory of Silkworm and Mulberry Genetic Improvement, Ministry of Agriculture and Rural Affairs, Sericultural Scientific Research Center, Chinese Academy of Agricultural Sciences, Zhenjiang 212100, China; 3 Institute of Animal Science, Jiangsu Academy of Agricultural Sciences, Nanjing 210014, China; 20220007@jaas.ac.cn (B.G.); liujie891213@163.com (J.L.); 4 Key Laboratory of Crop and Livestock Integration, Ministry of Agriculture, Nanjing 210014, China

**Keywords:** Yangzhou geese, monochromatic light, immune response, melatonin, fabricius

## Abstract

Light color is a key environmental factor regulating poultry immunity, but research data on geese remains limited. This study aimed to evaluate the effects of monochromatic light on the development of immune organs, antioxidant capacity, and immune response in Yangzhou geese. In this study, 240 male Yangzhou geese (4 replicates × 15 birds) were randomly assigned to white (400–700 nm), green (560 nm), blue (480 nm), or red (660 nm) light treatments for 70 days. In comparison to the white light (WL), both green light (GL) and blue light (BL) significantly enhanced the indices of thymus and bursa of Fabricius and upregulated proliferating cell nuclear antigen (PCNA) expression. These treatments also resulted in elevated serum levels of the anti-inflammatory factor IL-10 and immunoglobulins, while concurrently suppressing pro-inflammatory cytokines. Moreover, exposure to GL and BL significantly upregulated the mRNA expression of melatonin membrane receptors, whereas red light (RL) activated the mRNA expression of nuclear receptors. Furthermore, antioxidant enzyme activities were significantly elevated under GL conditions, along with a decreased malondialdehyde (MDA) content, in comparison to those under WL. These results indicate that GL and BL significantly enhance immune response and antioxidant defense in Yangzhou geese, whereas RL exerts inhibitory effects.

## Simple Summary

Preliminary investigations suggest that exposure to monochromatic green light enhances growth in Yangzhou geese at 70 days of age. However, the impact of light color on the development of immune organs, antioxidant capacity, and immune response remains unverified by conclusive research. In this study, 240 Yangzhou geese were randomly allocated into four treatment groups (*n* = 60 per group), with each group further divided into four replicates (15 geese per replicate). During the experimental period, the geese were exposed to a photoperiod consisting of 16 h of light and 8 h of darkness, with the light conditions being white light (WL), green light (GL), blue light (BL), or red light (RL) over a span of 70 days. Serum cytokine and immunoglobulin concentrations, immune organ indices, immune organ morphology, proliferating cell nuclear antigen (PCNA) expression in thymus and bursa of Fabricius cells, antioxidant capacity in the thymus and bursa of Fabricius, and melatonin receptor expression levels were assessed. The results demonstrated that exposure to green and blue light significantly enhanced immune responses and antioxidant capacity in Yangzhou geese, whereas red light exerted an opposing effect.

## 1. Introduction

Light is a critical environmental factor in poultry production, with its wavelength (color), intensity, and photoperiod playing significant roles in influencing growth performance [1]. In large-scale poultry farming, monochromatic light has become a key tool for optimizing production efficiency due to its controllable wavelength and high stability. Notably, specific light colors at particular wavelengths modulate growth performance by regulating feeding behavior and metabolic processes [2]. For example, green and blue light have been shown to improve growth performance by enhancing average daily gain (ADG) and feed conversion ratio (F/G) in chickens, while red light exerts an inhibitory effect [3,4,5]. Specifically, light exposure of varying wavelengths, intensities, and durations can significantly influence chicken growth performance through multiple physiological mechanisms, including regulating hormone secretion, affecting immune system function, and promoting musculoskeletal development [6]. However, research on the effects of monochromatic light remains inconsistent. Some studies have reported that exposure to red light leads to increased body weight compared to other monochromatic lights in poultry, such as Japanese quail [7]. Our preliminary investigation revealed that exposure to green light significantly increased the body weight of Yangzhou geese at 70 days of age, whereas red light exposure led to a reduction in average daily feed intake [8], demonstrating an opposite effect. Thus, the impact of light color on poultry growth performance demonstrates considerable interspecific variation and environmental dependency.

In addition to its impact on poultry performance, light exposure plays a significant role in modulating immune function through both direct and indirect mechanisms [9]. Photostimulation directly modulates immunity through three primary mechanisms: firstly, it promotes morphological maturation of central and peripheral immune organs (thymus, spleen, and bursa of Fabricius) while enhancing immune cell activity [10]. Specifically, monochromatic green light accelerates broiler thymus development by upregulating PCNA [11]; whereas toxins or stress reduce PCNA, which in turn causes bursal atrophy and impaired humoral immunity [12]. Secondly, blue and green wavelengths improve vaccine efficacy by boosting humoral immune responses and antibody production [13]. Thirdly, photoregulation suppresses pro-inflammatory cytokine synthesis, with green light being particularly effective at reducing avian inflammatory markers in chickens [14,15]. Therefore, most studies have focused on the relationship between light color and growth performance, while research on the association between light color and immune function has primarily centered on broiler chickens and laying hens. Studies on the Yangzhou goose, a dominant waterfowl breed in China, remain extremely scarce. The effects of light color on the morphology of immune organs, antioxidant defense mechanisms, and immune responses remain unclear.

Melatonin is an indole hormone with both antioxidant and immunomodulatory properties, and studies have confirmed that it plays a significant role in mediating the regulation of avian immune and antioxidant capacities by monochromatic light [16]. Melatonin, as a core mediator in light-color regulation, regulates immune cell proliferation and cytokine secretion through membrane receptors and nuclear receptors [17]. On the one hand, melatonin promotes the proliferation of LPS-stimulated B-lymphocytes by binding to the membrane receptors Mel1a and Mel1c on B-lymphocytes, activating the crosstalk between Gi/PI3K/AKT and PKC/ERK signaling pathways, and then regulating downstream GSK-3β and β-catenin. On the other hand, it can mediate the B-lymphocyte proliferation induced by the combination of green and blue monochromatic light by regulating the antioxidative capacity [9]. This indoleamine hormone plays a critical role in immune regulation, modulating the proliferation and differentiation of immunocytes along with immunoglobulin dynamics [18]. Notably, green and blue light upregulate the mRNA expression of Mel1a in chickens, thereby enhancing the proliferative capacity of immunocytes and overall immune competence [19]. However, systematic reports on the differential responses of melatonin receptors in Yangzhou geese to different light colors, and how these responses mediate antioxidant capacity and immune responses in the thymus and bursa of Fabricius, remain lacking.

The above findings provide supporting evidence for the regulatory role of light exposure in poultry immune function; however, due to species-specific differences, these results may not be directly applicable to geese. Therefore, this study systematically investigated the immunomodulatory effects of monochromatic light on Yangzhou geese, with a particular emphasis on the antioxidant capacity and humoral immune responses. It aims to establish a scientific foundation for optimizing light management strategies to enhance the production efficiency of Yangzhou geese.

## 2. Materials and Methods

### 2.1. Ethics Statement

All of the experiments and animal care were approved by the Ethics Committee of the Jiangsu Academy of Agricultural Sciences and followed the rules set on 8 July 2014 (Decree No. 63).

### 2.2. Animals and Treatments

Yangzhou geese were procured from Jiangsu Guiliu Animal Husbandry Group Co., Ltd. (Xuzhou, China). The experimental diets were formulated by the company to achieve the nutrient levels presented in Table 1. The feeding experiment was conducted at Liuhe Experimental Base of Jiangsu Academy of Agricultural Sciences. A total of 240 one-day-old male Yangzhou geese, with comparable body weights, were randomly allocated into four groups (60 per group, each consisting of four replicates with 15 geese per replicate). The geese were then exposed to white light (WL, 400–700 nm), green light (GL, 560 nm), blue light (BL, 480 nm), or red light (RL, 660 nm) via strips of LEDs (NVC Lighting Co., Ltd., Guangzhou, China) for 70 days. The temperature within the goose housing was maintained at 32 ± 2 °C for the initial 10 days, after which it was gradually adjusted to ambient room temperature. The experimental photoperiod was set at a light:dark ratio of 16:8 (6:00 on, 22:00 off). All light sources were equalized at an illuminance of 40 ± 5 lx at the geese’s head level using a Testo 540 illuminometer (Testo SE & Co. KGaA, Lenzkirch, Germany). Each group was separated by shading material to prevent light interference. The light-shielding material primarily consisted of a double-layer PET black and white film with a thickness of 0.26 mm, with the black film oriented inward and the white film outward. The light sources were calibrated every 10 days. From day 1 to day 20, the stocking density was maintained at 15 birds per square meter, which was subsequently reduced to 7.5 birds per square meter from day 21 to day 70. During the experimental period, the goslings were provided with ad libitum access to feed and water.

### 2.3. Growth Performance

The assessment of growth performance adhered to the methodology outlined in our previously published study [8]. Each morning prior to the first feeding (07:00), the remaining feed in the trough was weighed and recorded per replicate. The average daily feed intake (ADFI) was then calculated by subtracting the weight of leftover feed from the total amount of feed provided. Bird body weight was measured on day 70. The feed conversion ratio (FCR) was calculated as the total feed consumed divided by the total body weight gain.

### 2.4. Sample Collections

At the end of the 70-day experiment, 10 geese were randomly selected from each group as a subsample (with 2–3 geese per replicate). Blood samples were collected, followed by decapitation slaughter. Blood was collected from the right-wing vein using 5 mL EDTA-coated tubes. Blood samples were then centrifuged to obtain serum for subsequent analysis of inflammatory cytokines and immunoglobulins. The thymus and bursa were weighed to calculate the organ index and were fixed in 4% paraformaldehyde for morphological examination. Organ index = organ weight (g)/body weight (kg) × 100%. Additional thymus and bursa samples were collected, immediately snap-frozen in liquid nitrogen, and stored at −80 °C for subsequent qRT-PCR analysis and antioxidant capacity assessment.

### 2.5. Hematoxylin–Eosin (H and E) and Immunohistochemical (IHC) Staining

The thymus and bursa of Fabricius tissues were fixed in 4% paraformaldehyde for a duration of 72 h, subsequently dehydrated using a graded ethanol series, rendered transparent in xylene, and embedded in paraffin to facilitate the preparation of tissue blocks. Sections with a thickness of 5 μm were then obtained utilizing a Leica RM2016 rotary microtome (Shanghai Leica Instrument Co., Ltd., Shanghai, China). For histological staining, including H&E and IHC, the paraffin sections underwent dewaxing using an environmentally friendly dewaxing solution, followed by dehydration in anhydrous ethanol and rehydration through a graded alcohol series. For the observation of histological structures, the paraffin sections were stained with H&E. In the case of IHC, antigen retrieval was conducted, followed by three washes with phosphate-buffered saline (PBS). Endogenous peroxidase activity was inhibited by incubating the sections in 3% hydrogen peroxide for 10 min, after which they were rinsed again with PBS. Non-specific binding sites were blocked using 3% bovine serum albumin (**BSA**) for 30 min, followed by an overnight incubation at 4 °C with a primary antibody against proliferating cell nuclear antigen (**PCNA**) (Servicebio, Wuhan, China, catalog no.GB12010, diluted 1:500). The sections were subsequently treated with a horseradish peroxidase (**HRP**)-conjugated goat anti-mouse IgG secondary antibody (Servicebio, catalog no.GB23301, 1:200 dilution) for 50 min at room temperature. The chromogenic detection was performed using 3,3′-diaminobenzidine (**DAB**) substrate (Wuhan Sevier Biotechnology Co., Ltd., Wuhan, China), and hematoxylin was employed for nuclear counterstaining.

Finally, the tissue sections underwent dehydration, clearing in xylene, and coverslip mounting for microscopic observation. Utilizing an IX73-DP74 microscope (Olympus Optical Co., Ltd., Tokyo, Japan), we conducted an analysis of tissue sections from six Yangzhou geese. For each staining method (H&E and IHC), 24 randomly selected slices were assessed, with three microscopic fields quantified per slice. In the H&E-stained sections, the area (in mm^2^) of 5 thymic lobules and bursal nodules per section was measured using ImageJ software (version 1.54g, Bethesda, MD, USA). For the quantification of PCNA via immunohistochemistry, we randomly selected 3 visual fields (0.2 mm^2^ per field at 40× magnification) per section and calculated the total number of PCNA-positive cells per unit area using ImageJ software.

### 2.6. Determination of Antioxidant Activity

For the analysis of antioxidant capacity, 1 mg samples of thymus or bursa of Fabricius tissue were homogenized in PBS (1:9 *w*/*v* ratio) containing two glass beads and employing a mechanical homogenizer maintained in an ice-water bath. The homogenate was subsequently centrifuged at 3000 rpm for 10 min, and the supernatant was collected for further analysis. Using a BioTek microplate spectrophotometer (BioTek Instruments, Winooski, VT, USA) and following the manufacturer’s protocol (Nanjing Jiancheng Bioengineering Institute Co., Ltd., Nanjing, China), we measured four oxidative stress parameters: total superoxide dismutase (**T-SOD**) activity, catalase (**CAT**) activity, total antioxidant capacity (**T-AOC**), and malondialdehyde (**MDA**) levels.

### 2.7. Enzyme-Linked Immunosorbent Assay

Serum samples were obtained through the centrifugation of whole blood and subsequently subjected to analysis for immunoglobulin and cytokine concentrations. The serum levels of **IgA**, **IgM**, and **IgG** were quantified utilizing commercial goose enzyme-linked immunosorbent assay (ELISA) kits (Beijing RuiDaHengHui Science & Technology Development Co., Ltd., Beijing, China), following the protocols specified by the manufacturer. The linear regression of the standard curve was highly correlated with the expected concentration, with a correlation coefficient R ≥ 0.9900. The detection range for IgA, IgM, and IgG in goose serum was 1.0–320 μg/mL, 1.0–2400 μg/mL, and 1.0–800 μg/mL, respectively. The intra- and inter-assay coefficients of variation were both less than 15%. Concurrently, the concentrations of pro-inflammatory cytokines, including tumor necrosis factor α (**TNF-α**), interferon γ (**IFN-γ**), and interleukin 6 (**IL-6**), as well as the anti-inflammatory cytokine interleukin 10 (**IL-10**), were quantified using goose-specific ELISA kits (Jiangsu Meimian Industrial Co., Ltd., Nanjing, China). The detection ranges of the goose ELISA kits were: TNF-α (8–260 ng/L), IFN-γ (3–80 ng/L), IL-6 (1–40 ng/L), and IL-10 (3–80 ng/L). All measurements were conducted in triplicate using a Bio Tek microplate spectrophotometer (Winooski, VT, USA), with absorbance readings at 450 nm to ensure data reliability.

### 2.8. Real-Time Reverse Transcription-Polymerase Chain Reaction (qRT-PCR)

Total RNA was extracted from frozen thymus and bursa of Fabricius samples (−80 °C) using TRIzol reagent (R401-01, Vazyme, Nanjing, China), followed by quantification and quality assessment with a Nucleic Acid Protein Detector (IMPLEN, Munich, Germany). Subsequently, 1 μg of total RNA was reverse-transcribed into cDNA using a reverse transcription kit (Cat# RR047A, Takara, Dalian, China). qRT-PCR amplification was performed in triplicate using the Magic SYBR Mixture (CW 3008 M, CWBIO, Beijing, China) on a Bio-Rad CFX96 system (Hercules, CA, USA), with GAPDH employed as the endogenous control. Primer sequences are listed in Table 2. Relative mRNA expression levels were calculated using the 2^−ΔΔCt^ method.

### 2.9. Statistical Analysis

All data are presented as mean ± standard error of mean (SEM). Normality was assessed using the Shapiro–Wilk test. One-way analysis of variance (ANOVA) was conducted using GraphPad Prism version 9.0 (GraphPad Software, San Diego, CA, USA), followed by Tukey’s post hoc multiple-comparisons test. Differences were considered statistically significant at *p* < 0.05.

## 3. Results

### 3.1. Effects of Different Monochromatic Light on Serum Cytokines and Immunoglobulin Content of Yangzhou Geese

**Figure 1:**
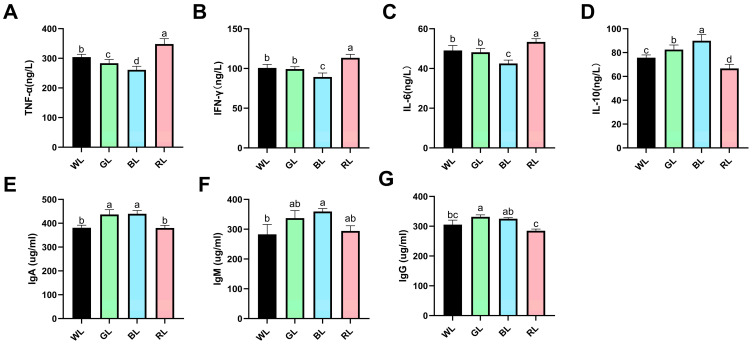
Effects of monochromatic light on serum cytokine and immunoglobulin levels in 70-day-old Yangzhou geese. (**A**) TNF-α, (**B**) IFN-γ, (**C**) IL-6, (**D**) IL-10, (**E**) IgA, (**F**) IgM, (**G**) IgG. WL: white light; GL: green light; BL: blue light; RL: Red light. Data are presented as means ± SEM. Distinct letters within the figure denote statistically significant differences (*p* < 0.05) among groups at 70 days of age.

To investigate the immunomodulatory effects of monochromatic light on Yangzhou geese, we conducted an analysis of its impact on serum inflammatory cytokines. Compared to the WL group, GL exposure resulted in a significant reduction in TNF-α levels, while BL treatment led to a downregulation of multiple pro-inflammatory cytokines, including TNF-α, IFN-γ, and IL-6 (Figure 1A–C). In contrast, RL exposure significantly increased all three pro-inflammatory markers, not only compared to the WL group, but also compared to the GL and BL groups. Moreover, both GL and BL exposures significantly elevated IL-10 levels compared to the WL group (*p* < 0.05), whereas RL treatment had the opposite effect, significantly suppressing this anti-inflammatory cytokine (Figure 1D).

Additionally, we measured serum immunoglobulin concentrations to assess humoral immune responses. Comparative analysis indicated that GL exposure significantly increased the levels of immunoglobulins IgA and IgG compared to the WL group (*p* < 0.05). BL exposure also significantly elevated the levels of immunoglobulins IgA and IgM compared to the WL group (*p* < 0.05). In contrast, RL exposure did not significantly affect immunoglobulin production. Notably, geese exposed to RL exhibited significantly lower serum IgA and IgG concentrations than those in the GL and BL groups (*p* < 0.05) (Figure 1E–G).

### 3.2. Effects of Different Monochromatic Light on Immune Organ Index of Yangzhou Geese

**Figure 2:**
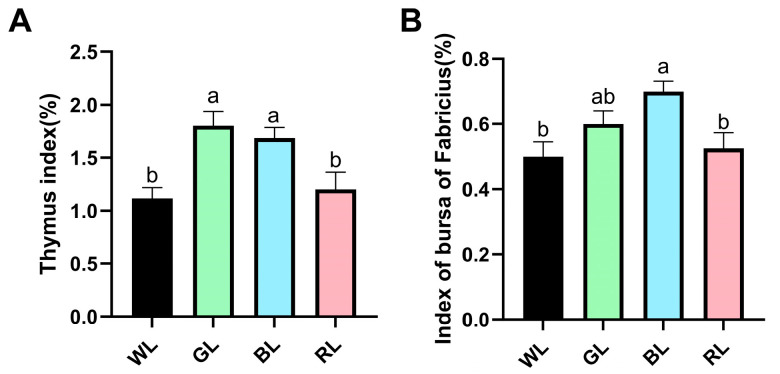
Impact of monochromatic light on the immune organ index of 70-day-old Yangzhou geese. (**A**) Thymus index. (**B**) Index of the bursa of Fabricius. The organ index is calculated as the ratio of organ weight (g) to body weight (kg). WL: white light; GL: green light; BL: blue light; RL: red light. Results are shown as the means ± SEM, with different letters indicating statistically significant differences (*p* < 0.05) among groups at 70 days of age.

Building on our previous findings that exposure to GL significantly increased the body weight of 70-day-old Yangzhou geese compared to WL or RL groups, the present study further explores the effects of monochromatic light on immune response by systematically assessing its impact on the immune organ index. Our results demonstrate that exposure to GL or BL significantly enhanced the thymus index in Yangzhou geese compared to the WL or RL groups (Figure 2A). Notably, the bursa of Fabricius index was markedly elevated in the BL group compared with the WL or RL group (Figure 2B). These findings indicate that GL and BL positively influence thymus development, while BL further promotes the growth of the bursa of Fabricius. In contrast, RL exhibited no statistically significant differences in the development of these immune organs.

### 3.3. Effects of Different Monochromatic Light on the Morphology of Immune Organs of Yangzhou Geese

**Figure 3:**
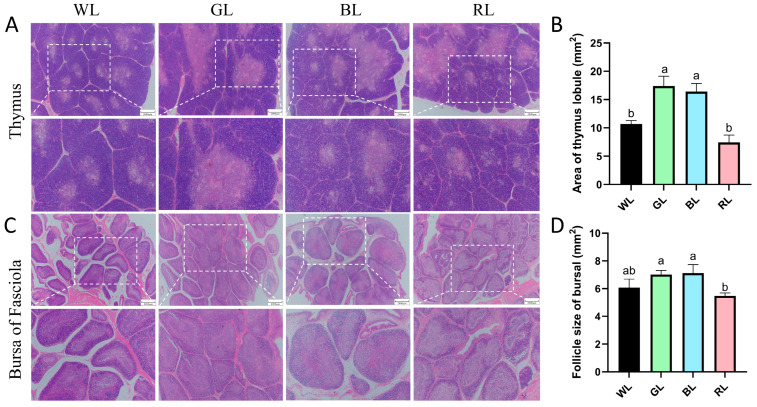
Effects of monochromatic light on thymus and bursa of Fabricius morphology in 70-day-old Yangzhou geese. (**A**) Structural morphology of the thymus. (**B**) Area of the thymus lobule. (**C**) Structural morphology of the bursa of Fasciola. (**D**) The area of bursa follicle. WL: white light; GL: green light; BL: blue light; RL: Red light. Data are presented as means ± SEM, with distinct letters indicating statistically significant differences among groups at 70 days of age. Scale bar: 2000 μm.

To evaluate the effects of monochromatic light on immune organ development, we analyzed the morphology of the thymus and bursa of Fabricius in 70-day-old Yangzhou geese using H&E staining. Quantitative analyses indicated that geese exposed to GL or BL exhibited significantly larger thymic lobule areas compared to those exposed to WL or RL (*p* < 0.05), with no significant difference observed between the WL and RL groups (Figure 3A,B). Similarly, in the bursa of Fabricius, nodule areas were significantly larger in the GL and BL groups compared to the RL group, while the RL group showed no notable difference relative to the WL group (Figure 3C,D). These morphological enhancements suggest that GL and BL irradiation during rearing can effectively stimulate the growth of immune organs, potentially enhancing systemic immunity in geese.

### 3.4. Impact of Various Monochromatic Light Exposures on PCNA Expression in Cell of Thymus and Bursa of Fabricius

**Figure 4:**
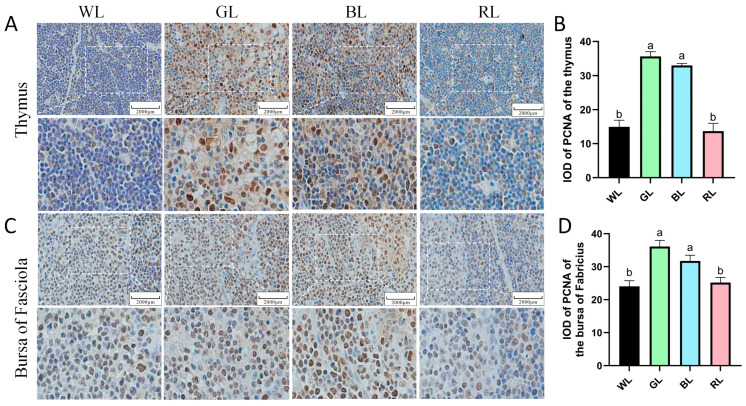
Monochromatic light effects on PCNA expression in immune cells of 70-day-old Yangzhou geese. (**A**,**B**) PCNA in the thymus. (**C**,**D**) PCNA in the bursa of Fabricius. WL, white light; GL, green light; BL, blue light; RL, red light. Data are presented as means ± SEM. The different letters marked on the figure represent significant differences (*p* < 0.05) from each other at 70 days of age. Scale bar: 2000 μm.

The proliferation activity of thymus and bursa of Fabricius cells in 70-day-old Yangzhou geese was assessed using IHC staining. PCNA-positive cells were predominantly localized in both the cortical and medullary regions of the thymus and bursa of Fabricius, indicating active cellular proliferation in these areas (Figure 4). Quantitative assessment revealed that exposure to GL and BL significantly increased the density of PCNA-positive cells (cells/mm^2^) in the thymus and bursa of Fabricius compared to the WL and RL groups (*p* < 0.05) (Figure 4A–D). These findings indicate that GL and BL substantially promote cellular proliferation in both thymic and bursa of Fabricius tissues, while RL exposure has no significant effects on immune cell proliferation.

### 3.5. Effects of Different Monochromatic Light on Antioxidant Properties of Thymus and Bursa of Fabricius

**Figure 5:**
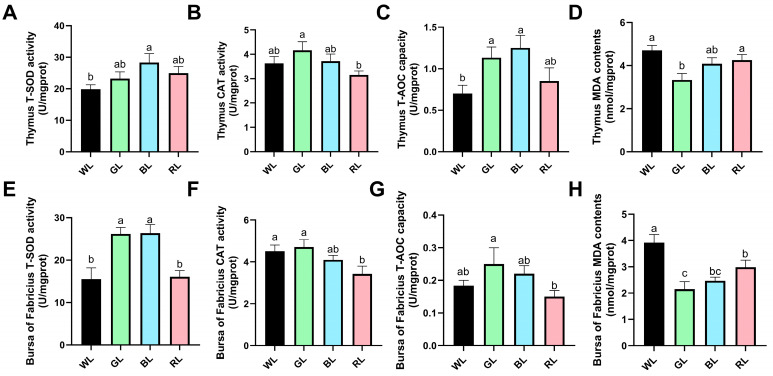
Effects of monochromatic light on antioxidant capacity in immune organs of 70-day-old Yangzhou geese. (**A**–**D**) Antioxidant status in thymus tissue: (**A**) total superoxide dismutase (T-SOD) activity, (**B**) catalase (CAT) activity, (**C**) total antioxidant capacity (T-AOC), and (**D**) malondialdehyde (MDA) content. (**E**–**H**) Corresponding antioxidant parameters in bursa of Fabricius: (**E**) T-SOD, (**F**) CAT, (**G**) T-AOC, and (**H**) MDA. WL, white light; GL, green light; BL, blue light; RL, red light. Data are presented as means ± SEM. The different letters marked on the figure represent significant differences (*p* < 0.05) from each other at 70 days of age.

In the thymus tissue, BL exposure significantly increased total superoxide dismutase (T-SOD) activity compared to those exposed to WL (*p* < 0.05) (Figure 5A), whereas GL and RL exhibited similar T-SOD levels and did not differ significantly from the T-SOD levels of the WL group. Notably, GL treatment significantly augmented catalase (CAT) activity relative to the RL group (Figure 5B), with BL showing intermediate effects similar to the WL group. Both GL and BL exposures significantly enhanced total antioxidant capacity (T-AOC) in thymus tissue compared to those exposed to WL (Figure 5C), while geese exposed to RL had no significant effect. Importantly, GL treatment significant decrease malondialdehyde (MDA) content compared to WL and RL (Figure 5D), suggesting superior oxidative stress mitigation among all treatments.

In the bursa of Fabricius, GL and BL exposures significantly enhanced T-SOD activity compared to the WL and BL groups (*p* < 0.05) (Figure 5E). Notably, CAT activity was significantly diminished under RL exposure compared to the WL and GL treatments (Figure 5F), whereas BL exposure exhibited no significant effect. Moreover, GL treatment significantly increased T-AOC activity in comparison to the RL group (Figure 5G). Concurrently, the content of malondialdehyde (MDA) in the bursa of Fabricius was significantly decreased under GL, BL, and RL exposure relative to the WL group (Figure 5H). However, no statistically significant differences were observed between the GL and BL groups. These coordinated responses underscore the unique ability of GL to enhance antioxidant defenses in bursal tissue.

### 3.6. Effects of Different Monochromatic Light on the Expression of Melatonin Receptor in Yangzhou Geese

**Figure 6:**
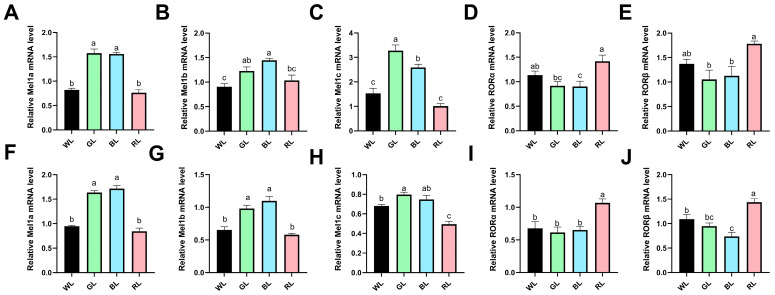
Monochromatic light modulates melatonin receptor gene expression in immune organs of 70-day-old Yangzhou geese. (**A**–**E**) Relative mRNA expression levels in thymus: (**A**) Mel1a, (**B**) Mel1b, (**C**) Mel1c, (**D**) RORα, and (**E**) RORβ. (**F**–**J**) Corresponding gene expression profiles in the bursa of Fabricius: (**F**) Mel1a, (**G**) Mel1b, (**H**) Mel1c, (**I**) RORα, and (**J**) RORβ. Light treatments: WL (white light), GL (green light), BL (blue light), RL (red light). Data are presented as means ± SEM (*n* = 6). The different letters marked on the figure represent significant differences (*p* < 0.05) from each other at 70 days of age.

Through the application of quantitative real-time PCR, we assessed the impact of monochromatic light on melatonin receptor expression patterns in the immune organs of Yangzhou geese. Our analysis yielded three principal findings: Firstly, in the thymus, GL and BL exposures significantly upregulated all three melatonin membrane receptors (Mel1a, Mel1c, and Mel1b) compared to the WL group (*p* < 0.05). While RL showed no significant effect, it should be noted that for Mel1b, there were no statistical differences between GL and RL treatments (Figure 6A–C). Secondly, the response of nuclear receptors exhibited an inverse pattern, with BL significantly reducing the expression of RORα in thymus tissue compared to the WL group. Moreover, geese exposed to RL significantly increased the expression of melatonin nuclear receptors RORα and RORβ in thymus tissue when compared to the GL and BL groups (Figure 6D,E). Thirdly, similar but not identical effects were observed in the bursa of Fabricius—GL and BL treatments significantly upregulated membrane receptors (Mel1a/b), while RL exposure did not exhibit a significant difference relative to the WL group (Figure 6F,G). Moreover, GL exposure significantly upregulated the expression of the membrane receptor Mel1c, whereas RL treatment specifically downregulated Mel1c expression compared to the WL group (Figure 6H). The nuclear receptor response in the bursa of Fabricius tissue partially mirrored the patterns observed in the thymus, with RL exposure substantially upregulating both RORα and RORβ, whereas BL treatment specifically reduced RORβ expression compared to the WL and RL treatments, though it did not differ significantly from GL (Figure 6I,J). These results indicate that: (1) green/blue and red light have opposing effects on melatonin signaling pathways and (2) membrane and nuclear receptors exert differential sensitivity to various light wavelengths.

## 4. Discussion

The retina of poultry comprises a variety of photoreceptors that exhibit high sensitivity to light across different wavelengths [20]. This characteristic has been harnessed in large-scale poultry farming, where artificial lighting is extensively utilized to enhance broiler productivity and mitigate seasonal reproductive constraints in Yangzhou geese. Among the critical parameters of light, its color has been demonstrated to significantly affect production performance and immune function in both broilers and laying hens [21,22]. Our previous research indicated that light color influences the productivity of Yangzhou geese, with green light notably increasing the body weight of 70-day-old geese. Building on these findings, it is imperative to further explore the effects of monochromatic light on their immune organ development, antioxidant capacity, and immune response. Such insights are vital for optimizing environmental light signals and improving the production efficiency of Yangzhou geese.

Immune organ indices, as a key immunological assessment metric, can objectively reflect the developmental state of the immune system and the overall immune function in poultry [23]. Previous studies have demonstrated that monochromatic light can affect the development of T lymphocytes in broilers [24], suggesting the critical role of light in influencing immune system development. Our study further reveals that exposure to green and blue light significantly increased the indices of the thymus and bursa organs, as well as expanded the morphological area of thymic lobules and bursal lymphoid nodules. These results corroborate the established role of light signaling, particularly green light, in promoting the development of immune organs [25]. Notably, the groups exposed to green and blue light exhibited a substantial increase in PCNA-positive cells within the thymus and bursa, supporting the hypothesis that specific light wavelengths enhance immune organ development by stimulating cellular proliferation. In contrast, the red light group did not show a significant difference in immune organ indices compared to the white light group. This lack of effect may be attributed to the inhibitory impact of red light on melatonin receptor Mel1a/c expression, potentially disrupting pathways involved in immune cell differentiation [26].

Melatonin receptors exhibit a tissue-specific distribution and expression profile, facilitating melatonin’s ability to mediate a variety of physiological effects via distinct receptor subtypes [27]. For example, the Mel1a receptor has been identified as a potential regulator of egg production in Yangzhou geese, while Mel1b and Mel1c receptors have been implicated in mediating melatonin’s role in promoting immune organ development and immune cell proliferation in broilers [28,29]. Notably, monochromatic light has been shown to modulate melatonin receptor expression, thereby influencing animal physiology. Previous research has indicated that green light upregulates the expression of membrane receptors while downregulating nuclear receptors in immune organs, in comparison to red light [30]. Our experimental results corroborate these findings: exposure to green and blue light significantly enhanced the mRNA expression of melatonin membrane receptors (Mel1a/b/c), whereas red light exposure suppressed Mel1a/c expression while activating nuclear receptors (RORα/β). This pattern of receptor expression is consistent with earlier studies conducted on broilers, supporting the proposed mechanism by which light signaling may regulate immune organ development through the modulation of melatonin receptors. Furthermore, melatonin exerts a protective effect against oxidative stress in immune organs by attenuating inflammatory responses [31]. This study provides further evidence that exposure to green light significantly enhances the antioxidant capacity of the thymus and bursa, thereby reducing oxidative damage, in accordance with previous findings [32].

TNF-α, IL-6, and IFN-γ are key biomarkers of inflammatory responses [31,33,34]. Our research demonstrated that exposure to green and blue light significantly downregulated pro-inflammatory factors (TNF-α, IFN-γ, and IL-6) while upregulating the anti-inflammatory cytokine IL-10 in Yangzhou geese. These results are consistent with previous studies by Guan et al. [35] and Chen et al. [36], which reported the anti-inflammatory effects of green light. In contrast, red light exposure increased levels of pro-inflammatory factors, potentially by inhibiting the melatonin signaling pathway.

Beyond cellular inflammatory factors, immunoglobulin levels serve as indicators of the body’s immune status. While earlier studies indicated that 14-day exposure to monochromatic light at 15 lx did not significantly affect IgY and IgM concentrations in goslings [37], our results revealed that at 40 lx, both green and blue light significantly increased serum IgY, IgM, and IgA levels. The findings indicate that immunoglobulin production is influenced not only by the color of light but also by its intensity. In broilers, adjusting the light intensity to an optimal level of 50lx from extreme levels (5 lx or 200 lx) resulted in a significant IgM response, underscoring the critical role of appropriate light intensity [31,38].

## 5. Conclusions

Overall, these findings suggest that exposure to blue or green light at optimal intensities can significantly improve the immune response in Yangzhou geese by simultaneously augmenting humoral immunity (through increased immunoglobulin levels) and cellular immunity (via anti-inflammatory effects and the development of immune organs). Moreover, melatonin receptor expression is affected by light color, suggesting a potential role in phototransduction and immune regulation. However, the specific mechanisms through which melatonin mediates light color-induced immunomodulation in the thymus and bursa of Fabricius remain to be elucidated.

## Figures and Tables

**Table 1:** Nutrient Levels of the Experimental Diets.

Composition	Content		
	d 1–d 15	d 16–d 45	d 46–d 70
Crude protein, %	18.0	17.0	17.0
Crude fat, %	6.0	9.0	11.0
Crude fiber, %	5.0	4.5	4.5
Calcium, %	0.8	0.85	1.0
Phosphorus, %	0.75	0.85	0.85
Metabolizable, Kcal/kg	2650	2850	3150

**Table 2:** Primers used in this study.

Genes	Primer Sequence (5′ to 3′)	Accession Number
Mella-F	GTTCCGCAGCTTCTTGTTG	XM_066996266.1
Mella-R	CTGGGTCACCTCCACCTTG	
Mel1b-F	TGTGGTAATTCATTTCATCGTCCC	U30609
Mel1b-R	TTGGTGCCATTTCTGAAGGATTGAT	
Mel1c-F	CAGATAAGTGGGTTCCTGATGGG	U31821
Mel1c-R	ACCGAAGGCTGTGGCAGATGTAG	
RORα-F	GGCAGTTATGCGCAGTCAAA	XM_067003930.1
RORα-R	TTCTGGGAGTCAAAGGCACG	
RORβ-F	GCAATGGCTTGAGCAACCTG	XM_048073568.1
RORβ-R	GCTGGGCAGAATCCACATTG	
GAPDH-F	GCCATCACAGCCACACAGA	XM_067004670.1
GAPDH-R	TTTTCCCACAGCCTTAGCA	

## Data Availability

The original contributions presented in this study are included in the article. Further inquiries can be directed to the corresponding authors.

## Abbreviations

The following abbreviations are used in this manuscript:

ADFI	average daily feed intake
AKT	Protein Kinase B
BL	blue light
BSA	Bovine Serum Albumin
CAT	catalase
DAB	3,3′-Diaminobenzidine
ERK	Extracellular signal-regulated kinase
FCR	feed conversion ratio
GAPDH	glyceraldehyde-3-phosphate dehydrogenase
GL	green light
GSK-3β	Glycogen synthase kinase-3 beta
Gi	Inhibitory G protein
H&E	hematoxylin and eosin staining
HRP	Horseradish Peroxidase
IHC	Immunohistochemistry
IgA	Immunoglobulin A
IgG	Immunoglobulin G
IgM	Immunoglobulin M
IFN-γ	Interferon-gamma
IL-10	Interleukin-10
IL-6	Interleukin-6
LPS	Lipopolysaccharide
MDA	malondialdehyde
Mel	melatonin membrane receptor
PBS	Phosphate-Buffered Saline
PCNA	Proliferating Cell Nuclear Antigen
PI3K	Phosphoinositide 3-kinase
PKC	Protein kinase C
qRT-PCR	quantitative real-time polymerase chain reaction
RL	red light
ROR	melatonin nuclear receptors
RORα	retinoic acid receptor-related orphan receptor α
RORβ	retinoic acid receptor-related orphan receptor β
T-AOC	total antioxidant capacity
TNF-α	Tumor Necrosis Factor-alpha
T-SOD	total superoxide dismutase
WL	white light
